# Adalimumab for orbital myositis in a patient with Crohn’s disease who discontinued infliximab: a case report and review of the literature

**DOI:** 10.1186/1471-230X-13-59

**Published:** 2013-04-04

**Authors:** Sanam Verma, Karen I Kroeker, Richard N Fedorak

**Affiliations:** 1 Division of Gastroenterology, University of Alberta, Zeidler Ledcor Center, Edmonton, Alberta, T6G 2X8, Canada

**Keywords:** Crohn’s disease, Ulcerative colitis, Orbital myositis, Extraintestinal manifestations, Inflammatory bowel disease, Infliximab, Adalimumab

## Abstract

**Background:**

Orbital myositis is a rare extra-intestinal manifestation of inflammatory bowel disease. Seventeen cases of Crohn’s disease associated orbital myositis and 3 cases of ulcerative colitis associated orbital myositis have been reported in the published literature since 1970. We report the use of adalimumab (Abbott, Canada, Inc.) for orbital myositis in a patient with Crohn’s disease who discontinued infliximab (Janssen, Canada, Inc.) and review of the published literature.

**Case presentation:**

A 35 year-old male with a 7-year history of Crohn’s disease was treated with an ileocolonic resection and re-anastomosis followed by infliximab which maintained full endoscopic and clinical remission for four years. After stopping the infliximab for infusion-related reactions he presented with 3-day history of severe right eye pain, pain with ocular movement, proptosis, and conjunctival injection. He had no intestinal symptoms and endoscopic assessment revealed no active luminal disease. CT of the orbit revealed an enlarged right medial rectus muscle with tendonous involvement and a diagnosis of orbital myositis was made. Treatment with 80 mg per day prednisone with tapering dose and adalimumab, induction and maintenance, resulted in rapid resolution of the orbital myositis and ocular symptoms with no recurrences on follow-up at 10 months.

**Conclusions:**

The current case demonstrates a rare extraintestinal manifestation of Crohn’s disease, orbital myositis, and its temporal relationship to the discontinuance of infliximab therapy and its successful treatment, without recurrence with tapering prednisone and adalimumab.

## Background

Extra-intestinal manifestations of Crohn’s disease (CD) are inflammatory manifestations that occur outside the gastrointestinal tract. Up to 30% of Crohn’s patients may present with multiple extraintestinal manifestations with the occurrence of one increasing the chances of others [1]. Ocular manifestations of CD occur infrequently with a prevalence rate of less than 10%; most of these cases being episcleritis and uveitis [2]. Orbital myositis (OM) is a very rare ocular extraintestinal manifestation of CD.

A literature review revealed that in a sample of 498 patients with Crohn’s only one patient had OM [3]. To determine the full published literature relating to OM as a complication of CD the PubMed and Medline databases were searched for relevant literature. Seventeen previous cases of Crohn’s related OM have been reported in literature. Only one previous study looked at adalimumab use in recurrent OM related to CD [4-9]. Therefore, in this patient group the use of adalimumab needs to be further explored.

## Case presentation

A 35-year-old Caucasian male was diagnosed with long segment ileal and extensive colonic CD in 2005, following a 10-year history of intermittent abdominal pain, cramps, and diarrhea. At diagnosis patient was placed on mesalamine (4 g/d), corticosteroids (prednisone 40 mg/d tapering over 12 weeks to zero) and azathioprine (150 mg/d) as induction therapy. He achieved clinical remission; however, within 8 months he developed an episode of small bowel obstruction and underwent an ileal resection with an ileocolonic anastomosis.

Post operatively he was treated with azathioprine 150 mg/d; however, markedly elevated liver enzymes led to the discontinuance of the azathioprine. Within 6 months of stopping the azathioprine he developed recurrent abdominal pain and diarrhea and colonoscopy confirmed anastomotic recurrence of his disease. He was then started on infliximab (5 mg/kg) monotherapy with an induction regime of 0, 2, 6 weeks followed by maintenance treatment every 8 weeks. He achieved a full remission, confirmed endoscopically, for four years.

After his 28^th^ infliximab infusion, he began to develop significant arthralgia and maculaopapular skin rashes on the dorsum of his hands and arms, which worsened after each infliximab infusion. There was no biochemical evidence of disease activity; hemoglobin, C-reactive protein, platelet count, serum iron and ferritin were normal. The infliximab was discontinued and endoscopic restaging and assessment for adalimumab insurance coverage was arranged. 6-mercaptopurine (75 mg/d) was started after discontinuation of the inflximab while the endoscopic restaging and assessment was pending. However, the 6-mercaptopurine was stopped after 3 weeks due to severe nausea and fatigue. The patient’s occupation prevented him from completing the restaging and insurance assessment and during this time he was on no therapy for his CD.

**Figure 1:**
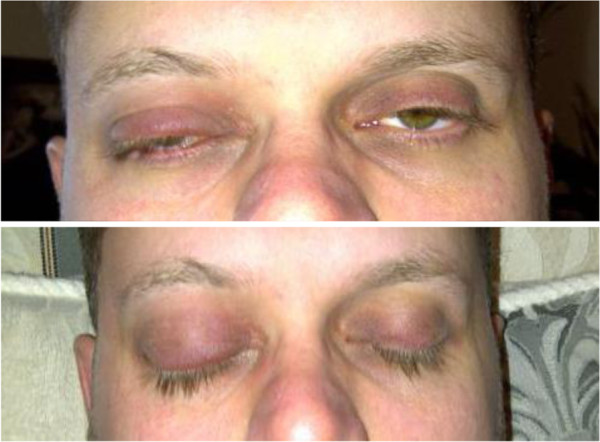
Patient at presentation with erythema and edema of the right upper eyelids, fullness of superior sulcus suggestive of proptosis.

**Figure 2:**
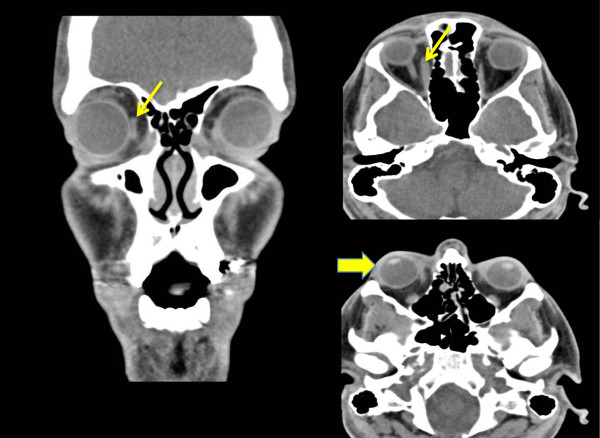
**Computerize tomography of the orbits revealed enlarged right medial rectus muscle with tendonous insertion involvement (small arrows) and mild proptosis (large arrow).** No fat stranding or scleral thickening was noted.

Six weeks after stopping the 6-mercaptopurine and 13 weeks after stopping the infliximab the patient presented to the emergency department with a three-day history of severe right eye pain. Examination revealed an uncorrected visual acuity was 20/25 OD and 20/20 OS. Intraocular pressure of the right eye was slightly elevated, the right upper eyelid was swollen and the conjunctiva injected (Figure 1). The right globe was 1.5 mm anterior than the left. Pupillary response to light was normal and there was no relative afferent pupillary defect. The patient had limited extraocular movement and significant pain on abduction and adduction of the right eye. No optic nerve abnormalities were noted, specifically no hyperaemia of the optic nerve or nerve fiber layer edema. The patient was afebrile and had normal hemoglobin, white blood cell count, C-reactive protein, iron studies and liver function studies. Computerize tomography of the orbits revealed enlarged right medial rectus muscle with tendonous involvement, mild proptosis, no fat stranding or sclerotic changes (Figure 2), and no sinus opacification was noted. The remaining physical exam was normal. Diagnosis of idiopathic orbital inflammatory syndrome, OM subtype, was made based on clinical findings and imaging. The patient was started on prednisone 80mg/d and tapered over 4 weeks. Within 48 hours there was improvement in the orbital pain, redness, and swelling and the patient was then started on adalimumab with induction dosing of 160 mg, 80 mg, followed by 40 mg weekly and methotrexate 12.5 mg once weekly as a standard combination therapy. Colonoscopy (after initiation of treatment of the OM) did not demonstrate active colonic or ileal disease. The OM resolved on the tapering prednisone and adalimumab and the patient has been asymptomatic on adalimumab for 10 months with no recurrence of the OM.

## Discussion

OM is a very rare extraintestinal manifestation of inflammatory bowel disease and is more common in CD than ulcerative colitis [10]. Seventeen previous cases of CD associated OM and three previous cases of UC related OM have been reported in literature. The clinical specifics related to these published case reports are summarized in Table 1.

**Table 1:** Published case reports (1976 – 2012) describing orbital myositis in patients with inflammatory bowel disease

**Study author/ year**	**Age/ gender**	**IBD (CD/UC)**	**Therpay at time of orbital myositis**	**Diagnostic test confirming orbital myositis**	**Treatment for orbital myositis**	**Response to treatment**	**Follow-up**
Bennion/2012 [10]	63 M	UC	Infliximab	CT	Infliximab methylprednisone	Resolved	13 months
Pimentel/2012 [9]	55 F	CD	Sulfasalazine	Gadolinium MRI	Infliximab prednisolone	Resolved	24 months
Hernandez-Garfella/2011 [6]	32 F	CD	Corticosteroids	MRI	Adalimumab	Resolved	36 months
Kondolt/2011 [11]	11 F	CD	None	CT MRI	Corticosteroid	Resolved	Unknown
Bourikas/2010 [7]	35 F	CD	None	MRI	Steroids	Resolved	12 months
Culver/2008 [4]	23 F	CD	Methotrexate Infliximab	Gadolinium MRI	Methylprednisone cyclophosphamide	1 recurrent episode	24 months
Ramahlo/2008 [12]	40 F	CD	Methotrexate 6-mercaptopurine	MRI	Prednisone	Resolved	Unknown
Leibovitch/2005 [8]	44 M	CD	Unknown	CT	Prednisolone oral antibiotics	Resolved	3 months
Macarez/2005 [13]	32 M	UC	Mesalamine	CT MRI	Corticosteroids	Resolved	12 months
Garrity/2004 [14]	34 F	CD	Prednisone	MRI	Infliximab methotrexate	Resolved	27 months
Garrity/2004 [14]	27 F	CD	Prednisone methotrexate 6 MP	Unknown	Infliximab	Resolved	27 months
Maalouf/2001 [15]	48 F	CD	None	CT	Prednisone	1 recurrent episode	96 months
Jain/2001 [16]	43 F	UC	Unknown	MRI	Corticosteroids	1 recurrent episode	8 months
Durno/1997 [17]	12 F	CD	None	MRI	Prednisone	3 recurrent episodes	Unknown
Squires/1992 [18]	20 M	CD	None	CT	Prednisone	1 recurrent episode	Unknown
Smith//1992 [19]	54 F	CD	Unknown	CT	Corticosteroid ileocolonic resection	2 recurrent episodes	12 months
Verbraeken/1984 [20]	38 F	CD	Sulfasalazine	Unknown	Coricosteroids Colectomy	Resolved	2.5 months
Weinstein/1984 [21]	17 F	CD	None	Unknown	Corticosteroids IV antibiotics	Resolved	6 months
Camfield/1982 [22]	15 F	CD	None	Unknown	Corticosteroids oral antibiotics ileocolonic resection	2 recurrent episodes	3 months
Greenstein/1976 [3]	Unknown	CD	Unknown	Unknown	Unknown	Unknown	Unknown

CT, computerized tomography; MRI, magnetic resonance imaging; CD, Crohn’s disease; UC, ulcerative colitis.

OM is inflammation of one or more extraocular muscles and generally presents with the mass effect of the inflammation, leading to orbital swelling, proptosis, diplopia, chemosis, pain, injection and opthalmoplegia [12,13]. The clinical presentation of CD-associated OM is similar to that of thyroid ophthalmopathy and as such, the pathogenesis needs to be determined early at presentation. Thyroid eye disease is typically bilateral, retracted eyelids, and has more involvement of the inferior and medial rectus while sparing tendonous insertions and appearing spindle shaped on imaging [10,23]. In contrast, in OM associated with CD there is a tubular configuration on imaging reflecting involvement of tendonous insertion [24]. Furthermore, thyroid ophthalmopathy usually does not cause pain as is the case in OM. The pathophysiology of CD associated OM remains unknown, but it has been hypothesized that immune complexes form to antigenic colonic mucoproteins and may cross react with extraocular muscles [21].

OM has been described to occur prior to the development of Crohn’s related gastrointestinal symptoms [7], and when the CD is in remission [9]. To our knowledge this is the first case that demonstrates OM occurring in a patient in clinical and biochemical remission after discontinuation of anti-TNFα therapy. The fact that the onset of the OM occurred 13 weeks after stopping infliximab is in keeping with the known infliximab pharmacokinetics [25].

Treatment of CD associated OM has traditionally involved corticosteroids as first line therapy, and low dose orbital radiotherapy, methotrexate, cyclosporine, and cyclophosphamide as second line therapies [26,27]. While the immediate inflammatory phase of OM is frequently corticosteroid responsive recurrences may occur once the dose is tapered [5]. Indeed, prior to the frequent use of immunosuppressive or anti-TNFα therapy for CD OM recurrence was common (Table 1). Since the patient in this case report presented with OM after discontinuation of infliximab, there was significant risk of recurrent OM once his corticosteroid treatment tapered to zero. Infliximab was used to successfully control chronic recurrent OM in 4 patients with inflammatory bowel disease (Table 1) and 3 non-inflammatory bowel disease patients who all failed standard treatment [14]. Adalimumab was successfully utilized to treat OM in a single patient with CD who had recurrent episodes and who failed to respond to standard immunosuppression medication (Table 1) [6] and in one non-inflammatory bowel disease-related case [5]. Mouse chimeric nature of infliximab may lead to the development of autoantibodies and result in blunting of the clinical response and development of adverse reactions. Adalimumab, with a fully humanized molecular structure may not elicit the same autoantibody response. The CARE study efficacy of adalimumab for extraintestinal manifestations of CD and found that there is no significant difference in EIM resolution based on previous infliximab exposure [28].

## Conclusion

The current case demonstrates a rare extraintestinal manifestation of CD, OM and its temporal relationship to the discontinuance of infliximab therapy and its successful treatment, without recurrence (for 10 months at the time of this publication), with tapering prednisone and adalimumab. Clinicians should be cognizant of this rare extraintestinal manifestation of CD that can occur in the absence of luminal disease activity, its propensity for recurrence and its successful response to anti-TNF*α* therapy.

## Consent statement

Written informed consent was obtained from the patient for publication of this Case report and any accompanying images. A copy of the written consent is available for review by the Editor of this journal.

## Competing interests

The authors declare that they have no competing interests (both financial and non-financial).

## Authors’ contribution

SV involved in drafting and conception of manuscript and acquisition of data. KK made substantial contributions to conception and design, analysis and interpretation of data and critical revision. RF made substantial contributions to conception and design, analysis and interpretation of data, critical revision, given final approval of the version to be published. All authors read and approved the final manuscript.

## Pre-publication history

The pre-publication history for this paper can be accessed here:

http://www.biomedcentral.com/1471-230X/13/59/prepub
